# The acceptability and clinical impact of using polygenic scores for risk-estimation of common cancers in primary care: a systematic review

**DOI:** 10.1007/s12687-024-00709-8

**Published:** 2024-05-21

**Authors:** Faye C. Dannhauser, Lily C. Taylor, Joanna S.L. Tung, Juliet A. Usher-Smith

**Affiliations:** 1 London, UK; 2 Department of Public Health and Primary Care, University of Cambridge, Cambridge, England

**Keywords:** Polygenic score, Systematic review, Primary care, Cancer, Risk stratification, Clinical utility

## Abstract

**Background:**

Polygenic scores (PGS) have been developed for cancer risk-estimation and show potential as tools to prompt earlier referral for high-risk individuals and aid risk-stratification within cancer screening programmes. This review explores the potential for using PGS to identify individuals at risk of the most common cancers seen in primary care.

**Methods:**

Two electronic databases were searched up until November 2023 to identify quantitative, qualitative, and mixed methods studies that reported on the acceptability and clinical impact of using PGS to identify individuals at highest risk of breast, prostate, colorectal and lung cancer in primary care. The Mixed Methods Appraisal Tool (MMAT) was used to assess the quality of included studies and a narrative synthesis was used to analyse data.

**Results:**

A total of 190 papers were identified, 18 of which were eligible for inclusion. A cancer risk-assessment tool incorporating PGS was acceptable to the general practice population and their healthcare providers but major challenges to implementation were identified, including lack of evidence for PGS in non-European ancestry and a need for healthcare provider education in genomic medicine. A PGS cancer risk-assessment had relatively limited impact on psychosocial outcomes and health behaviours. However, for prostate cancer, potential applications for its use in primary care were shown.

**Conclusions:**

Cancer risk assessment incorporating PGS in primary care is acceptable to patients and healthcare providers but there is a paucity of research exploring clinical impact. Few studies were identified, and more research is required before clinical implementation of PGS can be recommended.

**Supplementary Information:**

The online version contains supplementary material available at 10.1007/s12687-024-00709-8.

## Introduction

Cancer is the second leading cause of death globally, accounting for 10 million deaths in 2020 (Ritchie et al. 2018). The commonest cancers, lung (LC), breast (BC), prostate (PC) and colorectal (CRC), together account for nearly half of all cancers. By 2040, worldwide cancer rates are projected to increase by 55% with the greatest increase in cancer burden being seen in low and middle income countries (LMIC) (International Agency for Research on Cancer 2023). 30-50% of cancers are thought to be preventable (World Health Organisation 2022) and this highlights the role for cancer prevention and screening.

UK data from 2015-16, showed that most cases of cancer (61%) were diagnosed by GP referral, 19% as hospital emergencies and only 6% detected by national screening programmes (Cancer Research UK 2018). Primary care, therefore, plays a crucial role in the timely diagnosis of cancer. However, patients often present with non-specific symptoms and complex symptomatology. There is a need to develop methods to support primary care to detect cancer early.

Polygenic scores (PGS) reflect an individual’s estimated genetic predisposition to complex common diseases such as cancer. PGS are calculated by the aggregation of hundreds-to-thousands of single nucleotide polymorphisms (SNPs) with weak-to-moderate cancer associations which are weighted according to effect size (Uffelmann et al. 2021). The overall scores for an individual are compared to the general population. PGS have therefore been identified as potential tools to make more precise and personalised predictions about cancer risk (Pharoah et al. 2002). Applications include the development of stratified health screening programmes and enhanced cancer risk-prediction tools (Huntley et al. 2023).

PGS have been developed for many common complex diseases, but they show wide variability in application and reporting. This poses a barrier to implementation in clinical care. At the time of writing, a search of the PGS Catalog (PGS Catalog 2023) reveals 120, 66, 56 and 22 PGS associated with BC, PC, CRC and LC respectively. PGS Reporting Standards have been developed (Wand et al. 2021) and the “Evaluation of Polygenic Score Applications” PHG Foundation report (Moorthie et al. 2023) has been written to aid researchers and commercial companies developing PGS to overcome some of these barriers.

As genome sequencing becomes more affordable, genomic testing in primary care will become increasingly accessible. However it is important that before PGS are incorporated into clinical practice, their acceptability and clinical utility are demonstrated (Moorthie et al. 2021). In the context of cancer risk-estimation, PGS have potential to add value to existing cancer risk models. However, to be clinically useful, superiority needs to be demonstrated against existing practice. The benefits of their use need to outweigh harms, the setting of their use defined, thresholds for intervention set and cost-effectiveness demonstrated (Usher-Smith et al. 2015)

Given the potential of PGS in cancer risk prediction, this review aims to establish the current evidence for the acceptability and clinical impact of available PGS to identify individuals at highest risk of BC, PC, CRC or LC in the primary care setting.

## Methods

The Preferred Reporting Items for Systematic Reviews and Meta-analysis (PRISMA) 2020 systematic review guidelines informed the development and reporting of this study (Page et al. 2021).

### Search strategy

Medline and EMBASE electronic databases were searched from 2000 to November 2023 using a combination of title and abstract search terms and MeSH terms incorporating breast, colorectal, lung or prostate cancer, polygenic score, primary care and outcome. The full search strategy is presented in supporting information, S1*.*

### Study selection

#### Inclusion criteria

- Peer-reviewed primary research (English language)
- Quantitative, qualitative, or primary mixed methods studies
- BC, PC, CRC or LC risk-assessments
- Primary care population or primary healthcare providers
- Intervention focused on PGS or SNP cancer risk-alleles
- Outcomes included clinical feasibility, utility and acceptability of the genetic risk tool

#### Exclusion criteria

- High-risk individuals (including those with monogenic cancer risk susceptibility genes, recurrent or relapsing cancer or current cancer diagnosis)
- Population-based health screening cohorts without specific relevance to primary care
- Conference abstracts, letters, correspondence, editorials, comments or reviews

One reviewer (FD) conducted the database searches, removed duplicates and reviewed title and abstracts of all citations. A second reviewer (JT) independently screened a random sample of all the citations (10%) for inclusion and discrepancies were resolved in a consensus meeting. At least two reviewers (FD, JU, LT, JT) reviewed all citations eligible for full text review. Studies excluded by all reviewers were considered ineligible and discrepancies were again resolved at consensus. Two reviewers (FD & JU) agreed the final citations for inclusion. Further citations were identified from alternative source searches, such as through reference lists, Google and peer recommendation.

### Quality assessment

Two reviewers (FD and LT) independently completed quality assessment for all eligible papers using the Mixed Methods Appraisal Tool (MMAT) (Hong et al. 2018), which is appropriate for assessing the quality of qualitative, quantitative and mixed-methods research. No studies were excluded based on quality. Discrepancies, of which there were only a few, were discussed at consensus meetings. The final quality assessment is presented in supplementary information, S2.

### Data extraction and synthesis

Data extraction was conducted by FD for all eligible studies using a standardized form developed and piloted with a small number of studies. It included author, year, country, cancer type(s), primary aim, sample size, demographic characteristics, study design, recruitment, type of PGS, PGS uptake, data collection and outcomes and is included in supplementary information, S3. The same terminology used in the original papers in relation to ancestry, race and ethnicity was also used in the results discussion.

Outcomes were assessed according to categories shown in Table 1.

**Table 1:** Summary of common outcomes reported in eligible articles

Outcome categories	
Overall	PGS characteristics • Number of SNPs incorporated into risk assessment tool • Cancer type • Tool integration with additional risk factors • Absolute risk vs comparative risk measures
General Practice population	• PGS test uptake • Motivations for testing • Barriers to testing • Understanding of the test/ informed decision making • Psychosocial impact • Results – sharing and delivery preference • Health behaviour impact • Cancer diagnosis
Healthcare providers (HCP)	• Attitudes and opinions • Knowledge and training • Impact on screening recommendations

*SNPs* single nucleotide polymorphisms

## Results

### Study selection

**Fig. 1:**
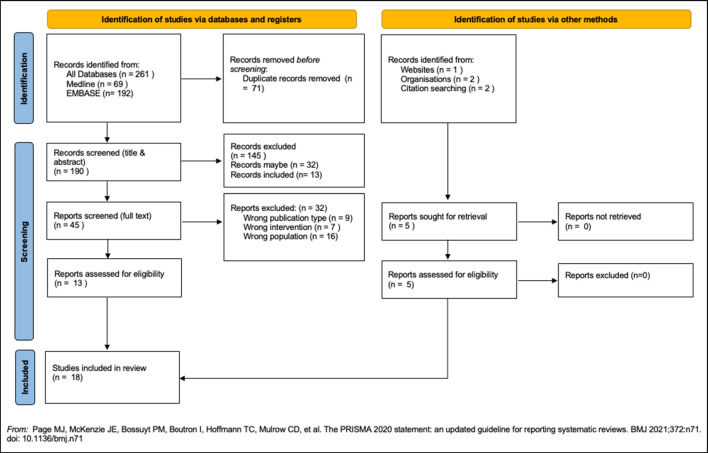
PRISMA Flow diagram

After removal of duplicates, the search generated 190 citations. Of these, 145 citations were excluded after title and abstract review. The commonest reason for exclusion being that the intervention did not include PGS. A further 32 citations were excluded after full text screening. An additional five papers were identified using alternative search methods, resulting in a total of 18 papers eligible for inclusion (Leventhal et al. 2013; Graves et al. 2013; Nusbaum et al. 2013; Conran et al. 2021; Saya et al. 2022; Saya et al. 2020; Butrick et al. 2014; Kirkegaard et al. 2018; Fredsoe et al. 2020a, 2020b; Green et al. 2022; Benafif et al. 2022; Kerman et al. 2023; Ayoub et al. 2023; Archer et al. 2020, 2023; Myers et al. 2015; Weinberg et al. 2014) (Fig. 1).

### Study characteristics

Variation was seen in study design and reported outcome measures, and this precluded meta-analysis or quantitative synthesis. The study characteristics and authors’ primary conclusions are summarized in Tables 2 and 3. Most studies explored the attitudes of patients to PGS testing (*n*=14, 78%), others explored HCP’s attitudes (*n*=4, 22%). Most studies considered PGS in relation to one cancer type (BC, PC or CRC). No eligible studies were identified for LC. Only four high-income countries were represented: eight USA (Leventhal et al. 2013; Graves et al. 2013; Nusbaum et al. 2013; Butrick et al. 2014; Conran et al. 2021; Kerman et al. 2023; Weinberg et al. 2014; Myers et al. 2015), five UK (Archer et al. 2020, 2023; Ayoub et al. 2023; Green et al. 2022; Benafif et al. 2022), three Denmark (Fredsoe et al. 2020a, 2020b; Kirkegaard et al. 2013) and two Australia (Saya et al. 2020, 2022). The majority of studies used an observational quantitative design (Graves et al. 2013; Saya et al. 2020; Fredsoe et al. 2020a, 2020b; Green et al. 2022; Benafif et al. 2022; Ayoub et al. 2023; Kerman et al. 2023; Conran et al. 2021; Weinberg et al. 2014), four used qualitative methods (Archer et al. 2020, 2023; Nusbaum et al. 2013; Kirkegaard et al. 2018) and four mixed methods (Leventhal et al. 2013; Saya et al. 2022; Butrick et al. 2014; Myers et al. 2015). Data were collected using demographic questionnaires, national surveys, baseline questionnaires, focus groups and semi-structured interviews. Three studies used case studies and simulated patients for HCP interpretation (Archer et al. 2020, 2023; Kerman et al. 2023).

**Table 2:**
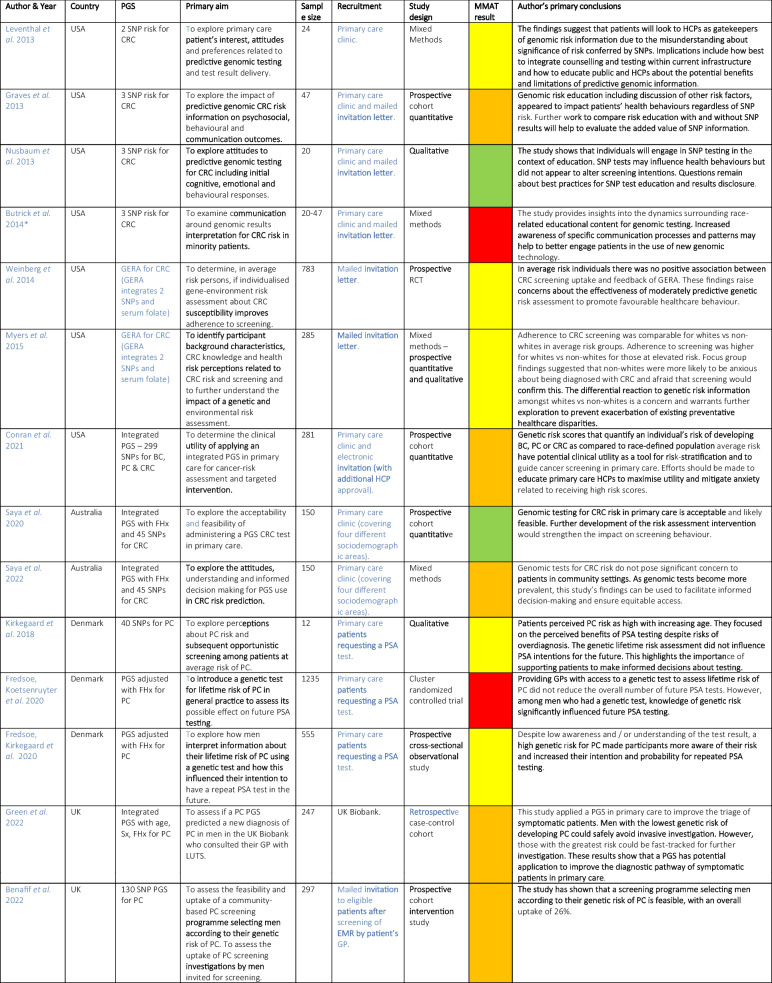
Study Characteristics and Author’s Primary Conclusions (Articles exploring patient’s attitudes to PGS)

*BC* breast cancer, *PC* prostate cancer, *CRC* colorectal cancer, *PGS* polygenic score, *SNP* single nucleotide polymorphism, *GERA* Gene Environmental Risk Assessment, *FHx* family history, *Sx* symptoms, *LUTS* lower urinary tract symptoms, *EMR* electronic medical record, *GP* General Practitioner, *HCP* Healthcare provider, *PSA* prostate specific antigen test; Green, ‘yes’ for all MMAT domains; Yellow, ‘can’t tell’ for one domain, Orange, ‘no’ for one domain or ‘can’t tell’ for two domains, Red, ‘no’ for two or more domains or ‘no’ for one domain and ‘can’t tell’ for one or more domains

*Study also included HCP’s attitudes

**Table 3:**
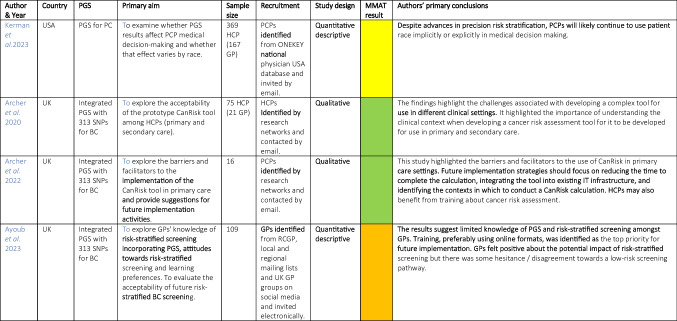
Study Characteristics and Author’s Primary Conclusions (Articles exploring HCP’s attitudes to PGS)

*HCP* healthcare provider, *GP* general practitioner, *PCP* primary care physician, *CPD* continuing professional development, *RCGP* Royal College Of General Practitioners

MMAT results: Green, ‘yes’ for all MMAT domains; Yellow, ‘can’t tell’ for one domain, Orange, ‘no’ for one domain or ‘can’t tell’ for two domains, Red, ‘no’ for two or more domains or ‘no’ for one domain and ‘can’t tell’ for one or more domains

## Data analysis

### Patient specific outcomes

#### PGS characteristics

Variations were seen in how PGS was calculated and how cancer risk was categorised, Table 4. Some studies used comparative measures of association (Conran et al. 2021; Green et al. 2022; Benafif et al. 2022; Weinberg et al. 2014; Myers et al. 2015) whilst others used absolute risk thresholds (Graves et al. 2013; Nusbaum et al. 2013; Saya et al. 2020; Butrick et al. 2014; Kirkegaard et al. 2018; Fredsoe et al. 2020a, 2020b; Archer et al. 2020, 2023). The number of SNPs used to calculate the risk score ranged from two to 313. Some risk scores were integrated with other health information. The CanRisk tool (CanRisk v2.3.5 2023) was the most comprehensive, integrating PGS with rare pathogenic risk variants and a wide range of non-genomic risk factors for breast cancer (Archer et al. 2020, 2023). The type of cancer, country of study and study type may have influenced these differences. Saya *et al.* (Australia) (Saya et al. 2020) converted their PGS into an absolute 10y risk in alignment with Australian screening guidance. In the UK, Green *et al.* (Green et al. 2022) categorized five risk thresholds between 1-5% for PC, in line with NICE guidance for suspected cancer referral where a referral is recommended for PPV ≥ 3% risk of cancer (National Institute for Health and CareExcellence 2015). In contrast the BARCODE1 pilot study (UK) defined high-risk for PC as individuals in the top 90^th^ centile conferring a 2.7 relative risk (Benafif et al. 2022). The studies by Fredsoe *et al.* (Denmark) (Fredsoe et al. 2020a, 2020b) expressed lifetime PC risk as either low or high. Finally, Archer *et al.* (UK) calculated lifetime or 10y risk of BC with thresholds defined as near population, moderate or high-risk (aligned with NICE guidance) (Archer et al. 2023). PGS variation meant that limited direct comparisons between studies could be made and a quantitative synthesis was not possible.

**Table 4:** Summary of PGS or risk score used in the different papers

Author	PGS	Categorization of PGS
Graves	3 SNP lifetime risk estimate (CRC)*	• Average risk: 10% estimated lifetime risk (for 2.5/6 SNP risk alleles) • High risk: ≥12% lifetime risk Possible range = 6% - 23%; sample range 6-15%; 20% participants were high risk
Nusbaum	3SNP lifetime risk estimate (CRC)*	• Average risk 6-8% • Moderate risk 9-11% • High risk ≥12% For the 20 participants estimates ranged from 6-14% lifetime risk CRC.
Butrick	3 SNP lifetime risk estimate (CRC)*	• Average risk: 10% estimated lifetime risk • High risk: ≥12% lifetime risk SNP scores for individual participants were not published.
Weinberg	2 SNP in combination with folate levels (CRC) **	• Average risk- identified by individuals with presence of average-risk alleles or high-risk alleles and normal serum folate • Elevated risk- identified by presence of high-risk alleles in combination with serum folate levels in lowest 25^th^ centile
Myers	2 SNP in combination with folate levels (CRC) **	• Average risk- identified by individuals with presence of average-risk alleles or high-risk alleles and normal serum folate • Elevated risk- identified by presence of high-risk alleles in combination with serum folate levels in lowest 25^th^ centile
Conran	299 PGS expressed as odds ratio value relative to average population risk (BC, PC and CRC)	• BC: low risk <0.5 to <1.0, average risk 1.0-1.4, high risk 1.5to >3.0 • PC: low risk <0.5 to <1.0, average risk 1.0-1.6, high risk 1.7to >3.0 • CRC: low risk <0.5 to <1.0, average risk 1.0-1.6, high risk 1.7to >3.0 Participants at high risk - BC 11.8%, PC 20.2% and CRC 6.1%. Participants at low risk - BC 62.0%, PC 66.0%, and CRC 56.9%. Subset of individuals with high risk PGS in addition to family history of cancer – BC 3.89%, PC 5.56 and CRC 1.83%.
Saya	45 SNPs combined with family history to generate PGS expressed as odds ratio then converted to absolute risk score (CRC) (AUC 0.63)	• Average risk ≤ 1% - no screening • Moderate risk >15 to ≤4% - iFOBT screen (immunochemical faecal occult blood testing) • High risk > 4% - colonoscopy Moderate risk participants 19.8% (25/126)
Kirkegaard	33 SNP lifetime risk after adjusting for family history (PC) †	• Normal risk < 30% lifetime risk PC • High risk > 30% lifetime risk PC Only participants with average risk PGS were included in the study.
Fredsoe/ Koetsenruyter	33 SNP lifetime risk after adjusting for family history (PC) †	• Normal risk < 30% lifetime risk PC • High risk > 30% lifetime risk PC Participants with normal risk 84.8%, high risk 11% and unknown risk 4.2% due to missing information.
Fredsoe/ Kirkegaard	33 SNP lifetime risk after adjusting for family history (PC) †	• Normal risk < 30% lifetime risk PC • High risk > 30% lifetime risk PC 13.2% participants were high risk and 86.8% were normal risk.
Green	269 SNPs weighted and aggregated to generate PGS expressed as relative risk (PC)	Incidence thresholds demonstrating relative risk within the population • 1^st^ quintile (1% incidence threshold)- lowest risk • 2^nd^ quintile (2% incidence threshold) • 3^rd^ quintile (3% incidence threshold)- NICE threshold for investigation • 4^th^ quintile (4% incidence threshold) • 5^th^ quintile (5% incidence threshold)- highest risk PGS +age model AUC 0.772 performed better than age or PGS alone. Adding FHx or symptomology led to negligible increase in AUC to 0.782
Benafif	130 SNP weighted risk-alleles aggregated - PGS expressed as relative risk score (PC)	• High risk PGS value = 11.5 (≥90^th^ centile) – MRI and prostate biopsy • Normal risk < 90^th^ centile- no PC screening PGS range 8.42-12.21 (median 10.34 and mean 10.33; SD 0.64). Participants with normal risk PGS 91%; high risk PGS 8.75% (referred for screening).
Archer 2020	313 SNP integrated model risk score (BC) used to generate lifetime risk and 10y risk ††	Integrated model includes multiple other lifestyle, hormonal, clinical risk factors and rare pathogenic variants. Risk aligns with NICE guidance. • High risk: ≥ 30% lifetime or ≥8% 10y risk – annual mammography from 30y • Moderate risk: ≥17% but <30% lifetime risk or ≥3 but <8% 10y risk- annual mammography from 40y • Near population risk: <17% lifetime risk or <3% 10y risk – mammography 3 yearly from 50y
Archer 2023	313 SNP integrated model risk score (BC) – used to generate lifetime risk and 10y risk ††	Integrated model includes multiple other lifestyle, hormonal, clinical risk factors and rare pathogenic variants. Risk aligns with NICE guidance. • High risk: ≥ 30% lifetime or ≥8% 10y risk – annual mammography from 30y • Moderate risk: ≥17% but <30% lifetime risk or ≥3 but <8% 10y risk- annual mammography from 40y • Near population risk: <17% lifetime risk or <3% 10y risk – mammography 3 yearly from 50y

*AUC* area under the curve, *MRI* magnetic resonance imaging, *NICE* National Institute of Clinical Excellence, *FHx* family history

Shared risk scores identified in groups - *, **, † and ††

#### PGS test uptake

Five publications considered the uptake of PGS testing (calculated as a percentage of participants who were offered the test and agreed to take it) for PC and CRC (Graves et al. 2013; Saya et al. 2020; Butrick et al. 2014; Benafif et al. 2022; Saya et al. 2022), Table 5. In all studies, test uptake rates were high, 84-96%. Despite race-specific limitations of testing in minority groups being explicitly mentioned within the study documentation, Butrick *et* al reported high uptake in a subgroup of minority patients, 95% (Butrick et al. 2014).

**Table 5:** – Summary of PGS uptake rates

Author	Country	Recruitment method	Participants race	Recruitment rate	PGS uptake rate
Graves*	USA	Waiting room and invitation letter	Caucasian 57% Multiracial 43%	30% (47/157)	96% (45/47)
Butrick*	USA	Waiting room and invitation letter	Caucasian 57% Multiracial 43%	30% (47/157)	96% (45/47) overall Caucasian (26/27) Multiracial (19/20)
Saya 2020 & 2022	Australia	Waiting room	Not specified	56% (150/264)	84% (126/150)
Benafif	UK	GP search of medical record and invitation letter	European only	26% (375/1436)	94% (307/328) (328/375 participants eligible and offered testing)

Shared participant recruitment *

#### Motivations for testing

Four studies reported on motivations for testing, Table 6*.* All studies reported that personal information gathering (Leventhal et al. 2013; Nusbaum et al. 2013; Saya et al. 2022; Kirkegaard et al. 2018) was a main reason for doing the test. Other reasons included gathering information for family members (Leventhal et al. 2013; Nusbaum et al. 2013), altruistic reasons (Leventhal et al. 2013; Nusbaum et al. 2013; Saya et al. 2022), on HCP’s recommendation (Leventhal et al. 2013) and due to personal risk factors or family history CRC (Leventhal et al. 2013; Saya et al. 2022). In the study by Kirkegaard *et al.* (Kirkegaard et al. 2013) participants were interested in knowing their PC risk but not specifically the genetic lifetime risk (Kirkegaard et al. 2018). Leventhal *et al.* reported that a subgroup of cancer survivors (*n*=5) were less interested in the test than non-cancer survivors (Leventhal et al. 2013).

**Table 6:** Summary of motivations for PGS risk assessment

Leventhal	• Information gathering (to know risk) • Implications for family • Altruism • Physician recommendation Survey: interest in how test changed risk (17%), due to personal risk factors or FHx of cancer (21%), know changes in cancer screening recommendations (13%) Cancer survivors (*n*=5) less interested in testing compared to non-cancer patients (χ^2^=7.14, *p*=0.03) expressed either disinterest or due to anticipated worry about test results.
Nusbaum	• Information gathering (irrespective of personal or family history of cancer) • Altruism (to contribute to research) • Curiosity • Implications for family • ‘Nothing to loose’ Most participants opted for testing despite knowing the uncertainty of the clinical utility of the test.
Saya et al. 2020	• Information gathering (so that something could be done about risk) • Reassurance about cancer risk • Family history of CRC • Altruism (to aid research / responsible thing to do) Cheek swab was viewed as straightforward and non-invasive. 60% Participants who were indifferent about the test still opted to take it
Kirkegaard	• Information gathering (to know overall personal cancer risk, not specifically genetic risk) • Early diagnosis of PC preference (even for indolent cancer type)

#### Barriers to testing

Three studies explored patient barriers to having a PGS test (Leventhal et al. 2013; Nusbaum et al. 2013; Saya et al. 2022), Table 7*.* Privacy and insurance concerns and the low perceived value of risk information were common to all (Leventhal et al. 2013; Nusbaum et al. 2013; Saya et al. 2022). Others included doubt that risk assessment would impact care (Leventhal et al. 2013), worry (Nusbaum et al. 2013; Saya et al. 2022) and a preference for knowing current risk rather than potential future risk (Saya et al. 2022).

**Table 7:** Summary of barriers to PGS for risk assessment

Leventhal	• Privacy / insurance concerns • Low perceived value (due to small influence on overall risk and focus on one disease) • Clinical utility doubts (due to uncertainty about impact on care) • Uncertainty about what to do with the results
Nusbaum	• Privacy / insurance concerns • Low perceived value (due to limited risk information from test) • Worry (however none felt that results would significantly increase personal distress)
Saya et al. 2022	• Privacy / insurance concerns • Low perceived value (due to lack of definitive information and availability of iFOB test) • Worry (not wanting to know something negative)

#### Patient understanding of the test / informed decision making

Four studies explored participant’s understanding of the genetic test (Fredsoe et al. 2020a; Saya et al. 2022; Saya et al. 2020; Nusbaum et al. 2013), Table 8*.* Notably, nearly half the participants in the study by Fredsoe *et al.* did not recall having a genetic test and significantly fewer high-risk individuals (75%) correctly identified their risk of PC compared to average-risk individuals (98.5%) (*p*<0.001) (Fredsoe et al. 2020a). The other three studies reported that participants generally understood the test (Nusbaum et al. 2013; Saya et al. 2022; Saya et al. 2020) with one reporting adequate knowledge in 83% participants (Saya et al. 2020). Saya *et al.* (Saya et al. 2022) reported that adequate knowledge was significantly higher in women, higher educated and primarily English speakers. Those participants opting for testing and higher educated individuals scored significantly higher on informed choice measures (Saya et al. 2022).

**Table 8:** Summary of patient understanding of PGS risk assessment (genetic test awareness, knowledge and informed decision making)

Nusbaum	Qualitative themes: • understood the gist of the information • Some difficulty with certain genomic concepts
Saya et al. 2020	83% participants (95% CI: 76-88%) had adequate knowledge of the test Better adequate knowledge seen in: • women (adjusted OR=3.05, 95% CI 1.12–8.54, *p*=0.030) • higher educated (adjusted OR = 4.51, 95% CI 1.67–12.98, *p*=0.004) • English primary language (adjusted OR = 6.06, 95% CI 1.30–31.76, *p*=0.024) No difference in knowledge between testers and non-testers (adjusted OR = 0.81, 95% CI 0.16–3.38, *p*=0.79).
Saya et al. 2022	Qualitative themes: Understanding risk indicated a “propensity to bowel cancer”. Higher knowledge scores were associated with: • more complex understanding of implications of test - understanding that PGS correlated with CRC but was not a direct cause of CRC • implications for screening recommendations MMIC (multidimensional measure of informed choice): • Informed choice made - 73% (95% CI: 65–80%) participants. • Testers more likely than non-tester to make informed choice (adjusted OR = 10.25, 95% CI 3.17–37.25, *p*=0.0002) • Higher educated made more informed choice (adjusted OR = 3.15, 95% CI 1.29–7.91, *p*=0.013) 47% non-testers may have been uninformed (positive attitude score for the test but chose not to take it)
Fredsoe/ Kirkegaard	Awareness of having a genetic test: • 55.1% responders (*n* = 306) recalled having a genetic test • Awareness of test did not differ between individuals with high versus normal risk (*p*=0.379) Correct identification of risk of PC: • Normal risk participants 98.5% • High risk participants 75% (*p*< 0.001) (Correct identification of abnormal PSA levels ≥4 μg/l: 51.7% of participants)

#### Psychosocial impact of the test

Six studies reported on the impact of having a PGS test on cancer worry and cancer related anxiety, reporting mixed results, Table 9*.* Three studies reported increased worry and distress (Myers et al. 2015; Conran et al. 2021; Saya et al. 2020). Myers *et al.* reported that for individuals at elevated risk of CRC, irrespective of race, participants had significantly increased worry and distress (Myers et al. 2015). Similarly, Conran *et al.* reported a significant increase in worry about risk of developing CRC in women and younger patients. Furthermore, those with a least one high PGS were significantly more worried about their risk of developing cancer (*p*=0.01) (Conran et al. 2021). Saya *et al.* reported less worry in patients with average-risk scores and significant increased cancer specific anxiety in patients with moderate-risk scores (*p*=0.01) (Saya et al. 2020). The qualitative study by Leventhal *et al.* noted a range of emotional responses (Leventhal et al. 2013). In contrast, the studies by Nusbaum *et al.* and Graves *et al. *(Graves et al. 2013; Nusbaum et al. 2013) reported no or low levels of distress or worry and no correlation between risk score and perceived risk (Graves et al. 2013). Nusbaum *et al.* reported that established risk factors such as personal or family history of cancer had a greater impact on perceived CRC risk (Nusbaum et al. 2013).

**Table 9:** Summary of the psychosocial impact on participants of PGS risk assessment

Author	Data collection	Findings
Leventhal	Focus group and survey	A range of emotional responses were reported to the test including disinterest, relief from knowing about risk and worry.
Graves	MICRA score (adapted)	Low levels of distress reported towards genomic testing (M = 5.4, SD = 3.8; possible Score Range 0 – 16). SNP risk scores were not related to perceived risk (*p*=0.08), CRC worry or genetic-testing distress post-test or at 3-month assessments (Multivariate analysis). Personal cancer history and family history of CRC were significantly associated with baseline perceived CRC risk (*p*<0.001).
Nusbaum	Semi-structured Interview	None of the participants expressed anxiety or distress during the post-test session or interview. SNP risk test results did not appear to have a substantial impact on perceived CRC risk. Individuals with personal or FHx cancer placed greater weight on established risk factors for CRC than on genomic information.
Myers	MICRA score and focus group	MICRA score significantly higher for white participants with elevated risk vs average risk (*p*=0.0338). MICRA score significantly higher for non-white participants with elevated risk vs average risk (*p*=0.0417).
Conran	US HINTS (US health information national trends survey) & Lerman’s CWS and CWS for genetic counselling.	Average age of participants with greatest, moderate, and least worry was 52.1y, 56.4y and 59.3y respectively (*p*=1.83E-4). Women reported significantly more worry with the proportion of women with greatest, moderate, and least worry being 88%, 72.7% and 61.5% respectively (*p*=0.01). High PGS scores were associated with anxiety. Proportion of participants with at least one high PGS expressing the greatest, moderate, and least anxiety were 88.9%, 56% and 17.3% respectively (*p*=3.76E-11). Individuals with at least one high PGS agreed significantly more strongly with the statement that they were worried about their risk of developing cancer (*p*=0.01).
Saya et al. 2020	CWS – difference at 1m and 6m after baseline and MICRA	Cancer worry: 3 groups (average PGS, moderate PGS or non-testers). Reduced worry in average risk group but no significant difference in worry in moderate risk or non-testers. Cancer specific Anxiety: At 1m the moderate risk group had significantly higher cancer specific anxiety compared to the average risk group (MICRA scores) 9.5, 15.9 respectively *p*=0.01.

*MICRA* multidimensional impact of cancer risk assessment– cancer specific anxiety, *CWS* cancer worry scale, *US HINTS* US health information national trends survey, *FHx* family history

#### Results delivery and sharing of information

Only two studies considered the method of results delivery (Leventhal et al. 2013; Nusbaum et al. 2013). Both indicated a preference for in-person delivery of results information. Participants in two studies (Nusbaum et al. 2013; Graves et al. 2013) frequently indicated intention to share results with family members but less frequently with HCPs.

#### Impact on health behaviours (future screening, PSA testing and lifestyle intent)

Eight studies reported on participant cancer screening intent, Table 10*.* Although four studies reported increased intentions for cancer screening (Conran et al. 2021; Graves et al. 2013; Nusbaum et al. 2013; Saya et al. 2020), objective measures of increased screening post-test were not seen in three studies (Graves et al. 2013; Weinberg et al. 2014; Saya et al. 2020). In contrast, one study by Benafif *et al.* demonstrated high prostate cancer screening uptake rates of 72% for individuals in the top 10% of PGS distribution (Benafif et al. 2022). Qualitative studies reported mixed views on screening intent (Leventhal et al. 2013) and differential views towards screening post-test between white versus non-white participants (Myers et al. 2015). Only two studies reported on lifestyle behaviour change, both reporting positive intent (Graves et al. 2013; Nusbaum et al. 2013).

**Table 10:** Summary of the impact of PGS risk assessment on future screening, PSA testing and health behaviour

Leventhal	• Mixed views on health habit or screening behaviour change • Individuals who were already doing everything to prevent cancer appeared less interested in the test • Individuals who perceived themselves to be at increased risk due to lifestyle factors felt the test would help motivate them to change behaviour and screening frequency
Graves	• At baseline 89% participants adherent to CRC screening guidelines vs 11% non-adherent (*n*=5) • Post-test 4/5 non-adherent participants intending to engage in screening • At 3 months 0/5 participants had taken up screening but 3/5 still intended to • Self-reported lifestyle change intent - 64% increased physical activity and 48% dietary change planned • At 3 months: 56% physical activity and 55% dietary change reported • SNP risk was not associated with change in physical activity or diet (multivariate logistic regression analysis)
Nusbaum	• 18/20 participants adherent to screening recommendations • 2/20 non-adherent. 2/20 intended to change screening intentions • At least half participants planned to decrease red meat and alcohol and increase physical activity • Some felt they could make little change due to already good health practices
Weinberg	• RCT – participants randomized to usual care vs genetic risk assessment group • Genetic test did not significantly improve screening adherence for CRC at 6 months post-test (adherence - usual care 35.7% vs genetic test 33.1%) • Overall screening adherence was not significantly different in average risk (38.1%) vs elevated risk (26.9%) groups (OR 0.75 CI 0.39-1.42) • Elevated risk vs average risk individuals – white participants were more likely to adhere to screening (adjusted OR 1.93 CI 0.56-6.69) • Elevated risk vs average risk individuals – non-white participants were less likely to adhere to screening (adjusted OR 0.38 CI 0.15-0.99)
Myers	• Differential response white vs non-white high-risk participants to results • High-risk participants: White 66.7% vs non-white 33.3% adherence to CRC screening • Non-whites concerned that screening would confirm the possibility of being diagnosed with CRC, whereas whites viewed screening as next step
Conran	• Participants already engaged in screening at baseline • individuals with ≥1 high risk PGS: screening intentions 60% increased, 15.8% stayed the same and 0% reduced (*p*=2.7e-8) • Younger patients indicated more likelihood of changing screening behaviour based on PGS results
Saya et al. 2020	PHM (preventative health model) used to measure mediators of screening at 6 months • Average risk group - attitudes to screening improved in 4/5 subscales (*p*=0.01-0.03) • Moderate risk group – attitudes to screening improved in 1/5 subscale (*p*=0.03) No difference was seen in risk-appropriate screening behaviour for genomic testers (64%) vs non-testers (54%) (OR = 1.5, 95% CI: 0.56–4.02, *p* = 0.37)
Fredsoe / Koetsenruyter	• RCT – Participating GP practices randomized to usual care (PSA test) vs intervention group (genetic + PSA test) Intervention vs control practices: • No significant difference overall in no. of PSA tests at 2y after adjusting for confounders (no. of PSA tests per 1000 men per practice, age, income) • No significant difference seen in no. of abnormal PSA tests Sub-group analysis: • Men with normal risk vs non-testers - lower chance of a future PSA test (OR = 0.62, *p* < 0.01) • Men with high genetic risk vs non-testers - higher chance of a future PSA test (OR = 8.94, *p* < 0.01) • Men with high genetic risk (6.6%) vs non-testers (2.2%) with elevated PSA test (OR = 3.31, 95% CI 1.57–6.97, *p* < 0.01)
Fredsoe / Kirkegaard	• High genetic risk score was the most significant predictor of a repeated PSA test (hazard ratio (HR) = 5.99; 95% CI = 4.09 to 8.79, *P*<0.001) • Median time to repeat PSA was 380 days (IQR 338 to 505 days) after the test. • Actual risk had strong positive correlation with intention to repeat the PSA test (*p*=2.2E-5)
Benafif	• 26 individuals in the top 10% of PGS distribution were offered prostate cancer screening with MRI + prostate biopsy • 7/26 men did not proceed with PC screening (1 died , 2 lost to follow up and 4 withdrew). Screening uptake for PC was 72%

Two studies reported on the number of repeat PSA tests done 2y after a PGS risk assessment for PC (Fredsoe et al. 2020a, 2020b). Overall there were no differences in PSA testing between the intervention and control practices even after adjusting for confounders (Fredsoe et al. 2020b). However subgroup analysis of the intervention practices showed that men with an average genetic risk had a significantly lower chance of having a future PSA test (OR = 0.62, *p*<0.01) and men with high genetic risk a higher chance of having a future PSA test (OR 8.94, *p*<0.01) when compared to men who did not have the genetic risk assessment (Fredsoe et al. 2020b). Furthermore a high genetic risk score was found to be the most significant predictor of a repeat PSA test (HR = 5.99, *p*=0.007) (Fredsoe et al. 2020a) and that higher percentages of elevated PSA test were found in men with high genetic risk compared with men who had no genetic test (OR = 3.31, *p*<0.01) (Fredsoe et al. 2020b).

#### Cancer diagnosis

Two publications studied the relationship between PGS and PC diagnosis (Green et al. 2022; Benafif et al. 2022)*.* Green *et al.* (retrospective study) determined incidence of PC within a 2y period for men presenting to their GP with lower urinary tract symptoms. They reported an association between the integrated PGS/age risk-score and incidence of PC with higher scores being seen in cases compared to controls (*p*=3.5e-30) (Green et al. 2022). They demonstrated that a PGS/age risk-score could be used to identify men at lowest risk of PC (<1% incidence of PC).

Benafif *et al.* (prospective study) used PGS to identify men at highest risk of PC. Men in the top 10% PGS score were offered screening with MRI and prostate biopsy. 8.75% of men were identified in this high-risk group. The PC diagnosis rate in this group was 26.9%, all 7 patients were placed under active surveillance programmes (Benafif et al. 2022).

### Primary healthcare providers attitudes and opinions

Four studies explored primary HCP’s attitudes to PGS for cancer risk assessment (Kerman et al. 2023; Ayoub et al. 2023; Archer et al. 2020, 2023), Table 11. All three studies which explored PGS in the context of a personalized BC risk assessment, demonstrated that HCPs attitudes were positive towards the concept but a need for education and training in genomics was highlighted (Archer et al. 2020, 2023; Ayoub et al. 2023)*.* Ayoub *et al.* reported that nearly half (49%) were not familiar with PGS and 23% reported that GPs were ‘not confident’ in explaining polygenic inheritance. A range of preferences for HCP learning formats were identified (Ayoub et al. 2023).

**Table 11:** Summary of knowledge and training needs of HCPs identified in the papers

Kerman	• The majority (90.8%) reported no additional genetics training beyond medical school
Archer et al. 2020	• Most HCPs felt confident in their communication skills around multi-factorial risk • Some HCPs were concerned about their limited genetics knowledge especially interpreting genetic risk • Lack of confidence in using the tool at point of care • Family history data input felt to be overwhelming for some • Lack of knowledge regarding prescribing risk-reducing medication • For nurses knowing when to hand over case to a GP colleague
Archer et al. 2023	• HCPs lacked confidence in BC knowledge, family history tools and risk management knowledge • Identified that good team communication and wider networks of support could increase confidence
Ayoub	• 49% HCP had no familiarity with PGS • Many lacked confidence in explaining polygenic inheritance, 10y risk score and pros/cons of stratification for screening • 13% specified that training would enhance implementation • Learning needs included: basics of risk stratification, PGS calculation and interpreting results • Learning format preferences: online courses 22%, website 18%, webinar-type conference 16%, in-person training 15%, phone or tablet App 11%, genetics specialist consultation 9%, printed material 7%

Archer *et al.* (Archer et al. 2020, 2023) reported on an integrated breast cancer risk assessment tool that incorporated PGS but there was little focus on the PGS component itself. Although participants reported confidence in communication skills regarding multi-factorial risk, they were concerned about their limited genetics knowledge and highlighted a need for additional training on interpreting genetic risk (Archer et al. 2020). Other more generalised concerns included the time needed for assessment, the importance of integrating the tool within clinical work flow and electronic medical record systems and the importance of providing clinical management information as part of the risk assessment (Archer et al. 2023).

Kerman *et al.* (Kerman et al. 2023) explored PC risk and reported that the majority of HCPs reported no additional genetics training beyond medical school.

Two studies considered use of a PGS tool in the context of ethnicity (Ayoub et al. 2023; Kerman et al. 2023). The study by Kerman *et al.* explored the influence of race on screening recommendations for PC and compared this to the patient’s risk using a PGS (Kerman et al. 2023). It was shown that physicians were more likely to recommend PC screening to black than white patients even for the same PGS (OR =1.58, *p*=0.025). There were no significant associations between the HCP’s self-reported race and ethnicity on PC screening recommendations. These results suggest that PGS did not remove the influence of race on clinical decision making.

In the study by Ayoub *et al.* (Ayoub et al. 2023) HCPs raised concerns that the use of a PGS tool that was validated in white European women would widen health inequality (Ayoub et al. 2023).

## Discussion

### Key findings

This review shows that the use of PGS to determine risk of common cancers in general practice is acceptable to patients and HCPs. High uptake rates of PGS testing amongst patients were seen despite concerns about privacy, insurance and limitations of testing in different ancestry groups. Patient acceptability is further supported by the relatively limited impact of PGS testing that was demonstrated on psychosocial outcomes. HCPs were also positive about the potential for PGS in cancer risk estimation. However, they highlighted a need for education and training in genomics and polygenic inheritance and raised concerns that using a PGS test with race-specific limitations could widen health inequality.

The impact on screening and health behaviour was limited. Although self-reported screening intentions were stated in several studies, this was not seen in objective outcomes. The impact of PGS testing for PC risk was explored in 3 studies which highlighted potential applications in primary care, including impact on future PSA testing rates (Fredsoe et al. 2020a, 2020b) and the potential to risk stratify individuals at risk of PC. (Green et al. 2022; Benafif et al. 2022).

### Strengths and limitations

A comprehensive search strategy with robust and clear definitions for key terminology was used including a range of terms for PGS. However, publications that used alternative terms for genetic risk, such as ‘CanRisk tool’ may have been missed. The search was limited to the top four most common cancers (BC, PC, CRC and LC). Excluding other cancer types appears justified given that no eligible studies were identified for LC. The adoption of a mixed methods approach enabled a comprehensive review of the current evidence available.

This review has several limitations. Firstly, there was a high degree of overlap seen in the eligible studies with 13 being published by five common research groups or patient cohorts (Butrick et al. 2014; Nusbaum et al. 2013; Graves et al. 2013; Leventhal et al. 2013; Weinberg et al. 2014; Myers et al. 2015; Kirkegaard et al. 2018; Fredsoe et al. 2020a, 2020b; Fredsoe et al. 2020a, 2020b; Saya et al. 2022; Saya et al. 2020; Archer et al. 2020, 2023), introducing potential source bias. Despite this, all studies were included as each reported on different qualitative and quantitative outcomes. Where indicated, overlap between studies have been highlighted within the tables, as seen for PGS uptake.

Secondly, all included studies were conducted in high-income countries and recruitment was often limited to Caucasian individuals (due to disparity in PGS accuracy across ancestry groups). This limits the transferability of these findings to LMICs and different ancestry groups. The lack of diversity in genetic studies is well recognised (Fatumo et al. 2022) and different methods are being developed to construct PGS from distinct ancestry groups to improve prediction and accuracy (Weissbrod et al. 2022; Ruan et al. 2022; Cavazos and Witte 2021). More balanced multi-ancestry genomic data sets are also being developed and include the ‘All of Us Program (USA)’ (National Institutes of Health (NIH) 2024), the ‘Million Veteran Program (USA)’ (US Department of Veterans Affairs (VA) 2024) and ‘Our Future Health (UK)’ (+ Our Future Health 2022).

Thirdly, the heterogeneity of the included studies meant we were unable to perform a quantitative synthesis or meta-analysis. Heterogeneity was seen across the study design and different cohorts as well as variations in the different uses of PGS and different outcomes. Differences were seen in the number of SNPs incorporated in the PGS and the way the PGS risk information was delivered to patients in some studies. This may have impacted individuals understanding and risk perception. It has been proposed that the communication of risk information is standardised to optimize interpretation and understanding (Brockman et al. 2021).

### Comparison with existing literature

Existing studies exploring PGS for risk-stratification in population-based cancer screening programmes demonstrate patient acceptability and are consistent with the findings in our study (Taylor et al. 2023; Laza-Vásquez et al. 2022). Overall public optimism was reported for risk-stratified screening programmes for breast, ovarian, kidney, prostate and all cancer types in a systematic review (Taylor et al. 2023) with the majority of studies related to risk-stratified breast and ovarian cancer screening. Similar acceptability was seen in the ‘DECIDO’ study for use of PGS to risk-stratify women for breast cancer screening (Laza-Vásquez et al. 2022). Although more research is needed in this area, the results from two large studies ‘WISDOM’ (USA) (Esserman 2017) and ‘MyPeBS’ hope to provide further evidence for acceptability for breast cancer risk-stratification incorporating PGS (MyPeBS 2022).

The absence of impact of PGS on screening and lifestyle behaviours seen in our study has also been seen in other common complex diseases including cardiovascular disease (Hollands et al. 2016; Silarova et al. 2019). Furthermore a review of systematic reviews found no evidence that presenting highly personalised risk information to individuals alone produced strong effects on lifestyle health-related behaviours (French et al. 2017).

The results presented here regarding HCPs attitudes to genetic cancer risk assessment are also consistent with existing literature. For example, a systematic review which explored clinicians’ attitudes to genetic cancer risk-assessment using family history tools in primary care (Laforest et al. 2019) showed that overall, general practitioners viewed their potential role in genetic risk assessment as important but found assessing risk difficult due to lack of knowledge and confidence in interpreting and explaining genetic test results. A further systematic review by Hamilton *et al* (Hamilton et al. 2017) explored primary HCPs knowledge and attitudes regarding genetic tests for individuals at risk of hereditary cancer syndromes and direct-to-consumer tests in relation to BC, PC and CRC. They highlighted a need for educational interventions due to low levels of genetic knowledge. Vassy *et al* (Vassy et al. 2023b) explored perceived benefits and barriers to implementing precision preventative care. Primary HCPs generally endorsed the use of PGS in preventative medicine but identified concerns regarding lack of understanding, potential patient anxiety and lack of evidence for use in non-European populations. In another study, similar barriers were seen in implementing risk-prediction tools for cancer in general (including those without genomic factors) (Usher-Smith et al. 2015).

### Implications for policy, practice (GP) and research

This review clearly demonstrates enthusiasm amongst HCPs and patients for PGS in cancer risk prediction, but further research and debate is needed before clinical application in general practice can be widely recommended.

The first priority is to develop and validate PGS in diverse populations. Although efforts are ongoing to improve performance of PGS across ancestry groups, the feasibility of using a population structure-adjusted PGS for multiple common diseases, including BC, PC and CRC, has recently been demonstrated (Hao et al. 2022). A further study “GenoVA study” (Vassy et al. 2023a) aims to use this tool to show how PGS might be equitably integrated into the busy primary care context whilst using a RCT trial design to compare the impact of PGS implementation versus usual care on patient outcomes. Risk assessment tools also need to be acceptable to hard-to-reach groups and for use in LMICs, thus ensuring equity of access.

Although the costs of genetic testing are falling and PGS cancer risk assessment tools have been shown to be acceptable to patients and HCPs in high income countries (HICs), these tools are unlikely to yet be a priority for LMICs (Pramesh et al. 2022). Very few LMIC have successful, sustainable cancer screening programs (Sullivan et al. 2021) with several barriers to implementation including sociocultural acceptance (Pak et al. 2021).

Currently most evidence exploring the potential of PGS for cancer risk-estimation is related to BC and PC. For BC risk-stratification using PGS, many large scale studies are underway including ‘MyPeBS’ (Europe) (MyPeBS 2022), ‘WISDOM’ (USA) (Esserman 2017) and ‘PROCAS’ (UK) (McWilliams et al. 2022). For PC the ‘ProCaRis’ study (University of Aarhus 2022) aims to determine if PGS can be used to better identify men at low risk of PC and reduce unsystematic PSA screening. Whilst preliminary results from the ’BARCODE1’ study have shown that PGS can be used to identify men at highest risk of significant PC (McHugh et al. 2022).

If primary HCPs are to play a pivotal role in genetic risk-assessment in the future, then the reported lack of knowledge and confidence surrounding genetic risk-assessment and interpretation highlights the urgent need for workforce training. In the UK, the government funded, NHS England Genomics Education Programme has developed a range of educational tools to upskill the workforce(Genomics Education Programme 2023). However global collaboratives will be needed to help support LMIC to participate in this space.

Further research should also focus on how to integrate genomic results with clinical decision support tools within the electronic healthcare record and to determine the best report format to use to aid risk communication with patients. This will enhance acceptability amongst HCPs. Results from the ‘eMERGE’ study, which aims to integrate genetic risk information with clinical care and lifestyle recommendations, may clarify how best to approach this (Linder et al. 2023).

Other innovations, such as multi-cancer early detection tests (MCEDs), show promise to support primary care to detect cancer early (Schrag et al. 2023) and the results of the NHS-Galleri clinical trial, using MCEDs in asymptomatic primary care populations, are awaited (Neal et al. 2022). Sensitivity of MCEDs has been shown to be poor though for some cancer types, including PC and BC (Klein et al. 2021), and this highlights the potential for a complimentary role for PGS particularly in the risk-assessment of PC and BC.

Finally, although PGS show promise and potential to improve risk prediction in cancer it is important that the focus on genetic risk does not detract from other contributors of disease such as environmental and lifestyle risks that may have potentially greater impact on cancer outcomes (Sud et al. 2023). For maximum benefit integrated PGS that include non-genomic risk factors should ultimately be used so that preventative strategies can be targeted at individuals at highest risk of cancer.

### Supplementary information

ESM 1 (PDF 111 kb)

ESM 2 (PDF 57 kb)

ESM 3 (XLSX 9 kb)

## Data Availability

No datasets were generated or analysed during the current study.
