# Supplementary material for: The acceptability and clinical impact of using polygenic scores for risk-estimation of common cancers in primary care: a systematic review
Source: J Community Genet. 2024 May 21;15(3):217–34. doi: 10.1007/s12687-024-00709-8 (PMC11217210; doi:10.1007/s12687-024-00709-8)
Supplement: Supplementary file 1 — (PDF 111 kb) [file 12687_2024_709_MOESM1_ESM.pdf]

Supplementary information:

S1 tables 1-3. Full Medline and EMBASE search strategies

**Objective:** To identify original research of the use of PGS in a primary care population to identify individuals at highest risk of developing breast or prostate or colorectal cancer or lung cancer.<br><br>**Data sources:** A systematic review of articles using Medline (1946 to November 27, 2023) and EMBASE (1974 to 2023 November 27). Search limited to 2000 onwards.<br><br>**Search strategy:** 69 searches Medline and 192 searches EMBASE<br><br>**Disease of Interest:** Breast cancer OR prostate cancer OR colorectal cancer OR lung cancer<br><br>**Population:** Primary care population<br><br>**Intervention:** Polygenic Score (PGS)<br><br>**Comparator / control:** not applicable<br><br>**Outcome:** clinical utility, early cancer detection, cancer risk, cancer prevention, feasibility, acceptability and cost-effectiveness.
**(S1) Table 1 Search Strategy**

**Ovid MEDLINE(R) ALL <1946 to November 27, 2023>**	**Search Terms**	**Results**
**1**	exp lung neoplasm/ or exp bronchial neoplasm/ or exp carcinoma, non-small cell/	279123
**2**	((lung or bronch*) adj3 (carcinoma* or cancer* or neoplasm* or tumo?r*)).mp.	371149
**3**	1 or 2	373763
**4**	exp breast neoplasm/	347543
**5**	(breast adj3 (carcinoma* or cancer* or neoplasm* or tumo?r*)).mp.	477138
**6**	4 or 5	477155
**7**	exp colorectal neoplasm/ or exp colonic neoplasm/ or exp rectal neoplasm/	241329
**8**	((colorectal or colon* or rect*) adj3 (carcinoma* or cancer* or neoplasm* or tumo?r*)).mp.	319613
**9**	7 or 8	330506
**10**	exp prostatic neoplasm/	150722
**11**	(prostat* adj3 (carcinoma* or cancer* or neoplasm* or tumo?r*)).mp.	199735
**12**	10 or 11	199735
**13**	3 or 6 or 9 or 12	1287157
**14**	((polygen* or genetic* or genomic*) adj2 (risk* or score*)).mp.	33564
**15**	((polygen* or genetic* or genomic*) adj predictor*).mp.	1205
**16**	exp Risk Assessment/	312642
**17**	exp Multifactorial Inheritance/	4164
**18**	16 and 17	191
**19**	((polygenic or multifactorial or complex or multigenic) adj (inheritance or trait* or character*)).mp.	14995
**20**	(risk adj2 (assessment* or analys*)).mp.	397784
**21**	19 and 20	356
**22**	(PRS* or PGS* or GRS*).mp.	29823
**23**	14 or 15 or 18 or 21 or 22	60355
**24**	exp General Practice/ or exp primary health care/ or exp family practice/	264079
**25**	((general adj practice) or (primary adj2 care) or (family adj (medicine or practice))).mp.	290843
**26**	24 or 25	386888
**27**	exp "early detection of cancer"/ or exp primary prevention/ or "patient acceptance of health care"/ or exp patient satisfaction/ or exp "attitude of health personnel"/ or cost-benefit analysis/ or exp cost-effectiveness analysis/	618064
**28**	(early adj (detection or diagnosis) adj2 cancer).mp.	42962
**29**	(cancer adj2 (risk* or prediction* or screening* or prevention*)).mp.	150440
**30**	((primary or primordial) adj2 prevention*).mp.	40511
**31**	(((health adj2 care) or healthcare) adj2 accept*).mp.	56245
**32**	(patient* adj2 satisfaction).mp.	122707
**33**	(clinical adj (utility or application or accuracy or value or validity)).mp.	125811
**34**	(prediction* adj2 accuracy).mp.	16621
**35**	(cost adj2 effectiveness).mp.	79294
**36**	(attitude* adj2 (staff or health personnel)).mp.	133162
**37**	((cost or economic) adj2 (analys* or evaluation*)).mp.	171231
**38**	27 or 28 or 29 or 30 or 31 or 32 or 33 or 34 or 35 or 36 or 37	1037152
**39**	13 and 23 and 26 and 38	74
**40**	limit 39 to yr="2000 -Current"	69

(S2) Table 2 Medline search

Embase <1974 to 2023 November 27>	Search Term	Results
**1**	exp lung tumor/ or exp lung cancer/ or exp lung carcinoma/ or exp bronchus tumor/ or exp bronchus cancer/ or exp bronchus carcinoma/	495359
**2**	((lung or bronch*) adj3 (carcinoma* or cancer* or neoplasm* or malignan* or tumo?r*)).mp. [mp=title, abstract, heading word, drug trade name, original title, device manufacturer, drug manufacturer, device trade name, keyword heading word, floating subheading word, candidate term word]	523616
**3**	1 or 2	597089
**4**	exp breast cancer/ or exp breast tumor/ or exp breast carcinoma/	657460
**5**	((breast or mammary) adj3 (carcinoma* or cancer* or neoplasm* or malignan* or tumo?r*)).mp. [mp=title, abstract, heading word, drug trade name, original title, device manufacturer, drug manufacturer, device trade name, keyword heading word, floating subheading word, candidate term word]	756566
**6**	exp breast tumor/	657460
**7**	4 or 5	764490
**8**	exp colon cancer/ or exp colon tumor/ or exp colon carcinoma/ or exp rectum cancer/ or exp rectum tumor/ or exp rectum carcinoma/ or exp colorectal cancer/ or exp colorectal tumor/ or exp colorectal carcinoma/	464366
**9**	(((colon adj3 rectum adj3 (cancer or malignan*)) or (colo-rectal or colorectal or recto-colonic or rectocolonic)) adj (cancer* or carcinogenesis or malignan*)).mp. [mp=title, abstract, heading word, drug trade name, original title, device manufacturer, drug manufacturer, device trade name, keyword heading word, floating subheading word, candidate term word]	269433
**10**	((colon* or mesocolon or coli) adj3 (mass or carcino* or neoplas* or tumo?r* or tumorigenesis or malignanc*)).mp. [mp=title, abstract, heading word, drug trade name, original title, device manufacturer, drug manufacturer, device trade name, keyword heading word, floating subheading word, candidate term word]	89636
**11**	((rect* or pararectal) adj4 (mass or neoplas* or tumo?r* or carcino* or malignanc*)).mp. [mp=title, abstract, heading word, drug trade name, original title, device manufacturer, drug manufacturer, device trade name, keyword heading word, floating subheading word, candidate term word]	44608
**12**	8 or 9 or 10 or 11	504140
**13**	exp prostate cancer/ or exp prostate tumor/ or exp prostate carcinoma/	299138
**14**	(prostat* adj2 (malignan* or cancer or tumo?r* or neoplas* or carcinoma)).mp. [mp=title, abstract, heading word, drug trade name, original title, device manufacturer, drug manufacturer, device trade name, keyword heading word, floating subheading word, candidate term word]	321655
**15**	13 or 14	326808
**16**	3 or 7 or 12 or 15	1937506
**17**	((polygen* or genetic* or genomic*) adj2 (risk* or score*)).mp.	102134
**18**	((polygen* or genetic* or genomic*) adj predictor*).mp.	2138
**19**	exp Risk Assessment/	754806
**20**	exp Multifactorial Inheritance/	270320
**21**	19 and 20	28394
**22**	((polygenic or multifactorial or complex or multigenic) adj (inheritance or trait* or character*)).mp.	16779
**23**	(risk adj2 (assessment* or analys*)).mp.	887029
**24**	22 and 23	563
**25**	(PRS* or GRS* or PGS*).mp.	44742
**26**	17 or 18 or 21 or 24 or 25	155235
**27**	exp general practice/ or exp primary medical care/ or exp family medicine/	219190
**28**	((general adj practice) or (primary adj2 care) or (family adj (medicine or practice))).mp.	390934
**29**	27 or 28	390934
**30**	16 and 26 and 29	256
**31**	exp early cancer diagnosis/ or exp primary prevention/ or exp patient attitude/ or exp "cost effectiveness analysis"/ or exp health personnel attitude/ or exp "cost benefit analysis"/	1003647
**32**	(Cancer adj2 (risk* or prediction* or screening* or prevention*)).mp.	397419
**33**	((primary or primodial) adj2 prevention*).mp.	66389
**34**	(((health adj2 care) or healthcare) adj2 accept*).mp.	1391
**35**	(clinical adj (utility or application or accuracy or value or validity)).mp.	172825
**36**	(prediction* adj2 accuracy).mp.	19295
**37**	(cost adj2 effectiveness).mp.	217187
**38**	(attitude* adj2 (staff or health personnel)).mp.	90501
**39**	((cost or economic) adj2 (analys* or evaluation*)).mp.	308029
**40**	31 or 32 or 33 or 34 or 35 or 36 or 37 or 38 or 39	1626936
**41**	30 and 40	203
**42**	limit 41 to yr="2000 -Current"	192

(S1) Table 3 EMBASE
