# Supplementary material for: The acceptability and clinical impact of using polygenic scores for risk-estimation of common cancers in primary care: a systematic review
Source: J Community Genet. 2024 May 21;15(3):217–34. doi: 10.1007/s12687-024-00709-8 (PMC11217210; doi:10.1007/s12687-024-00709-8)
Supplement: Supplementary file 2 — (PDF 57 kb) [file 12687_2024_709_MOESM2_ESM.pdf]

Supplementary information

S2 MMAT consensus meeting outcomes

**Qualitative Studies**						
Author	*Appropriate approach?*	*Adequate methods?*	*Findings derived from data?*	*Substantial interpretation?*	*Coherence?*	
Nusbaum 2013	y	y	y	y	y	
Kirkegaard 2018	y	?	y	Y	y	
Archer 2020	y	y	y	y	y	
Archer 2023	y	y	y	y	y	

**Quantitative RCT**						
Author	*Appropriate randomisation?*	*Baseline groups comparable?*	*Complete outcome data?*	*Blinding of assessors?*	*Adherence to intervention?*	
Weinberg 2014	y	y	y	?	y	
Fredsoe / Koetsenruyter	y	n	y	?	y	

**Quantitative non-randomised**						
Author	*Represent target population?*	*Appropriate measurements?*	*Complete outcome data?*	*Confounders accounted for?*	*Intervention administered as intended?*	
Conran 2021	n	y	y	y	y	
Graves 2013	n	y	y	y	y	
Saya 2020	y	y	y	y	y	
Benafif 2022	y	y	y	n	y	
Green 2022	n	y	y	y	y	
Fredsoe / Kirkegaard	?	y	y	y	y	

**Quantitative descriptive**						
Author	*Relevant sampling strategy?*	*Representative sample?*	*Appropriate measurements?*	*Non-response bias?*	*Appropriate statistical analysis?*	
Ayoub 2023	y	?	y	?	y	
Kerman 2023	y	y	y	?	y	

**Mixed Methods**						
Author	*Adequate rationale?*	*Integrated components?*	*Adequate output interpretation?*	*Inconsistencies addressed?*	*Quality of components?*	
Saya 2022	y	y	y	y	n	
Meyers 2013	y	y	y	y	?	
Butrick 2013	y	n	y	y	n	
Leventhal 2013	y	y	y	y	?	

Screening questions (for all types):
S1 Are there clear research questions?
S2 Do the collected data allow to address the research questions?

All studies passed the screening questions S1 and S2

Yes (y) scored in all MMAT domains	
Can't tell (?) scored for up to 1 domain	
No (n) scored for up to 1 domain *or* can't tell (?) for 2 domains	
No (n) scored for 2 or more domains *or no* (n) for one domain *AND* can't tell (?) for 1 or more domain	
